# Drug Shop of Galen

**Published:** 1846-09

**Authors:** 


*Drug Shop of Galen.* By Dr. Hamilton.—On the way to the Fever Hospital or the Hospital S. Giovanni, we passed through the Forum and among the numerous temples, palaces, triumphal arches, the amphitheatre and other colossal ruins which distinguish this portion of the ancient city, one humble spot, more than all others will remain for ever sacred in my memory; and to this spot alone did my feet repeatedly turn, as more than once I attempted to follow my guide and interest myself in the more stately ruins about me. You may smile at my peculiar romance, but if ever you visit Rome, go along the Forum to where stands upon the Via Sacra the ruins of the celebrated temple of Concord. Close by, in the days of Commodus, stood the drug shop of old Galen, for so he tells us himself. You will not see his sign, nor the illuminated colored bottles at the windows, for the shop was destroyed in a great conflagration, at the same time with the temple, but if you remember well the life of this excellent father and skilful physician, you will turn to mark the place and perhaps linger thereabouts for a while musing, and thinking possibly the old man will come to look after his broken bottles and his plasters and ointments which he prized so highly. For to judge from the amount of broken walls and loose stones laying about, the fire may have occurred but yesterday.

Galen had enjoyed when he came to Rome in the year 165 the rare privilege of examining a couple of human skeletons at Alexandria, and furnished thus with an extraordinary knowledge of anatomy, he gave public lectures upon the subject to the citizens of Rome. He had thereby attained great celebrity both as a physician and surgeon, when the jealousy of rival physicians procured his banishment from the city, to which he did not return until recalled by Aurelius, after whose death he was made physician to the young and profligate Emperor Commodus. He lived long afterwards to enjoy his restoration to fame and fortune, having died at the advanced age of 70 years.—*Buffalo Med. Jour., Aug.* 1846.

